# Artificial Adaptive and Maladaptive Sensory Receptors Based on a Surface‐Dominated Diffusive Memristor

**DOI:** 10.1002/advs.202103484

**Published:** 2021-11-27

**Authors:** Young Geun Song, Jun Min Suh, Jae Yeol Park, Ji Eun Kim, Suk Yeop Chun, Jae Uk Kwon, Ho Lee, Ho Won Jang, Sangtae Kim, Chong‐Yun Kang, Jung Ho Yoon

**Affiliations:** ^1^ Electronic Materials Research Center Korea Institute of Science and Technology (KIST) Seoul 02791 Republic of Korea; ^2^ Department of Materials Science and Engineering Seoul National University Seoul 08826 Republic of Korea; ^3^ Department of Materials Science & Engineering Korea Advanced Institute of Science and Technology (KAIST) Daejeon 34141 Republic of Korea; ^4^ Department of Materials Science and Engineering Korea University Seoul 02841 Republic of Korea; ^5^ KU‐KIST Graduate School of Converging Science and Technology Korea University Seoul 02841 Republic of Korea; ^6^ Department of Nuclear Engineering Hanyang University Seoul 02841 Republic of Korea

**Keywords:** adaptation, diffusive memristors, maladaptation, metal‐oxide nanorods, nociceptors, receptors, threshold switching

## Abstract

A biological receptor serves as sensory transduction from an external stimulus to an electrical signal. It allows humans to better match the environment by filtering out repetitive innocuous information and recognize potentially damaging stimuli through key features, including adaptive and maladaptive behaviors. Herein, for the first time, the authors develop substantial artificial receptors involving both adaptive and maladaptive behaviors using diffusive memristor. Metal‐oxide nanorods (NR) as a switching matrix enable the electromigration of an active metal along the surface of the NRs under electrical stimulation, resulting in unique surface‐dominated switching dynamics with the advantage of fast Ag migration and fine controllability of the conductive filament. To experimentally demonstrate its potential application, a thermoreceptor system is constructed using memristive artificial receptors. The proposed surface‐dominated diffusive memristor allows the direct emulation of the biological receptors, which represents an advance in the bioinspired technology adopted in creating artificial intelligence systems.

## Graphical abstract

Adaptive and maladaptive biological receptors serve as the interface between external information and the inner nervous system in all vertebrates. A new class of artificial receptors is developed using surface‐dominant diffusive memristor. Unique switching dynamics allow them to mimic the adaptive and maladaptive operation with key parameters of the sensory receptor, including short‐latency, selective response, and tunability of adaptation rate.

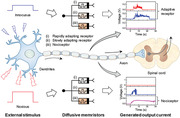

## 1 Introduction

A sensory receptor is a fundamental element specialized in detecting changes in external information and trigger impulses in the sensory nervous system.^[^ ^1^, ^2^, ^3^ ^]^ It is located ubiquitously in the human body and serves as the interface between external information and the inner nervous system in all vertebrates, allowing humans to sense, perceive, and interact with the environment. In this sensory system, the receptor is only triggered by an external stimulus that exceeds a specific threshold value, followed by adaptation or maladaptation to prolonged stimulus.^[^ ^4^, ^5^ ^]^ These biological functions play a crucial role in enabling a living system better suited to the environment. Based on the adaptation rate, sensory receptors are primarily classified as rapid, slow, and non‐adapting receptors. Adaptive receptors are triggered by innocuous stimuli that exceed a threshold value and adapt by decreasing their sensitivity, enabling organisms to filter out irrelevant repetitive information.^[^ ^6^, ^7^ ^]^ In contrast, the maladaptive receptor is triggered with a high threshold value, higher than innocuous stimuli, and does not adapt to noxious stimuli, excessively intense and results in tissue damage, for conscious awareness of pain, the so‐called nociceptor.^[^ ^8^, ^9^, ^10^ ^]^ Therefore, it is of great importance that organisms react differently to whether the stimulus is noxious or not.

Bioinspired electronics are technologies that mimic the sensory transduction of biological receptors from an external stimulus to an electrical signal, signal transmission of biological neurons, and synaptic plasticity.^[^ ^11^, ^12^, ^13^, ^14^, ^15^, ^16^ ^]^ A memristor has successfully demonstrated the potential to mimic leaky integrate‐and‐firing (LIF)^[^ ^17^ ^]^ in biological neurons and synaptic functions including the transformation of short‐term plasticity to long‐term plasticity,^[^ ^18^, ^19^ ^]^ spick‐timing‐dependent plasticity, and spick‐rate‐dependent plasticity.^[^ ^20^, ^21^ ^]^ Although these qualitative functionalities of the neuron and synapse may represent significant steps toward the realization of bioinspired electronics, electronic sensory receptors with all features of threshold, adaptation, and maladaptation functions have not yet been implemented. Therefore, incorporating artificial receptors into the electronic nervous system can potentially replace malfunctioning organs and create humanoid robots that have fine sensory systems, such as ours.^[^ ^22^, ^23^ ^]^ In addition, output signals from an artificial receptor can be conjugated as an input stimulus for neuromorphic computing.^[^ ^24^, ^25^, ^26^ ^]^

Recently, diffusive memristor has been reported as bioinspired electronics, which are essentially volatile threshold switching devices with switching dynamics of ionic migration (Cu or Ag) across an insulating layer (SiO_2_, HfO_2_, or Ta_2_O_5_).^[^ ^27^, ^28^, ^29^ ^]^ Intrinsic threshold switching, in which a voltage larger than the threshold, induces a transition from a high‐resistance state (HRS) to a low‐resistance state (LRS) makes them the most suitable candidate to mimic the threshold in biological receptor.^[^ ^30^, ^31^ ^]^ However, these devices inevitably require a large diffusion barrier for active metals in a dense oxide matrix, resulting in an electroforming process and a long‐latency.^[^ ^32^, ^33^ ^]^ In biological systems, the latency of channel opening is extremely short (less than 40 µs).^[^ ^34^ ^]^ Furthermore, to the best of our knowledge, there have been no reports on substantive artificial receptors involving both adaptive and maladaptive behaviors to prolonged stimuli. A few studies have reported fragmentary characteristics of artificial receptors, most of which have implemented only maladaptive (nociceptive) characteristics. Therefore, to realize diffusive memristor‐based artificial receptors, the memristor must fulfill the requirement of “short‐latency” between a stimulus and the response it triggers, “selective response” referring to as a response of adaptation in an innocuous stimulus and of maladaptation in a noxious stimulus, and “tunability” of electrical characteristics for adaptive and maladaptive operations.

**Figure 1:**
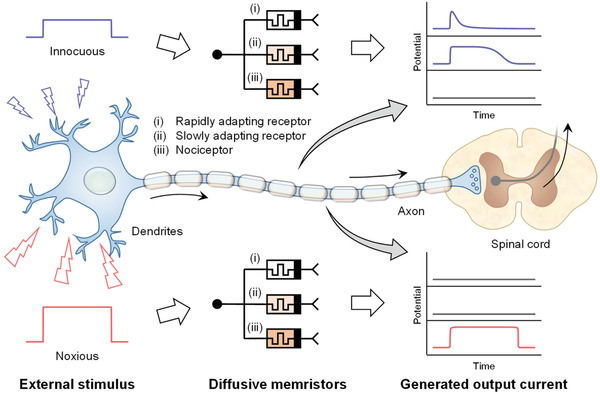
Concept illustration of artificial receptors based on diffusive memristors. Working principle of biological sensory nervous system using fully diffusive memristor‐based artificial receptors. The adaptive memristors are triggered by an innocuous stimulus followed by rapid and slow adaptation. When a noxious stimulus is received from a maladaptive nociceptor, the output current is generated without adaptation.

Herein, we introduce artificial receptors using diffusive memristors based on the surface migration of Ag on SiO_2_ nanorods (NRs). In a two‐terminal stack structure, metal‐oxide NRs as a switching matrix provides an opportunity to engineer the device characteristics. The active metal can be preconfigured on the surface of porous oxide NRs during fabrication and migrate rapidly along the surface under electrical stimulation, inducing electroforming‐free and short latency, respectively. The electrical characteristics of the threshold switching device are closely related to the formation of a conductive filament (CF) in the active switching area.^[^ ^19^, ^35^ ^]^ For the metal‐oxide NR‐based memristor, the CF formed on the surface can be finely controlled from thin to thick diameter through the amount of active metal, inducing a competing effect between electromigration and Joule heating. Under prolonged stimulation, the thin CF is ruptured by subsequent Joule heating, whereas the electrical on‐state is maintained for the thick CF. Hence, the adaptive and maladaptive switching behavior can be implemented based on the structural features. **Figure** **1** illustrates sensory information processing from external stimuli to the spinal cord and brain. The perception of a stimulus stems from the response of a specific receptor located at the end of the sensory neuron's axon. The response of the nervous system depends on the history of stimulation, and different types of receptors adapt to prolonged stimulation differently, such as rapid, slow, and no adaptation. In the diffusive memristor‐based nervous system, the adaptive memristors are turned on (to LRS) to innocuous stimuli, followed by rapid and slow adaptations (to HRS). In contrast, the maladaptive memristor, the so‐called nociceptor, responds only to noxious stimuli without adaptation because of a relatively higher threshold for conscious awareness of pain. This process corresponds to perception with “selective response” and “tunability” characteristics in the bio‐system and was demonstrated here for the first time using surface‐dominated diffusion memristors.

## 2 Results and Discussions

**Figure 2:**
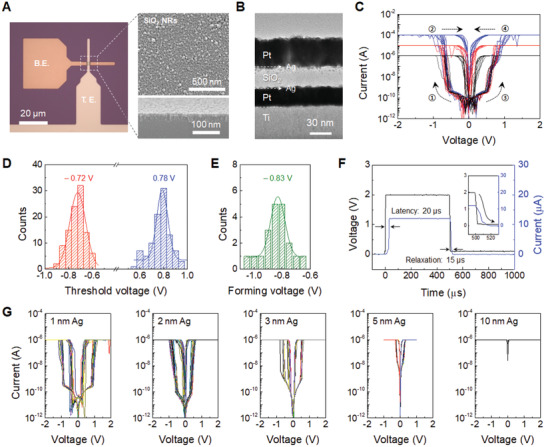
Memristor structure and threshold switching characteristics. A) Optical and SEM images of a cross point diffusive memristor and porous SiO_2_ NRs. B) Cross‐sectional TEM image of a diffusive memristor with a stack of Pt/Ag/SiO_2_ NRs/Ag/Pt. C) Typical *I*–*V* curves for the threshold switching under DC voltage sweeps with various compliance currents. Histogram for D) Threshold voltage distribution and E) Electroforming voltage distribution. F) Latency characteristic of the device and the inset shows the relaxation behavior. G) Electrical switching *I*–*V* curves with different amount of Ag from 1 to 10 nm.

The optical image in **Figure** **2**A shows a cross‐point diffusive memristor device with a junction size of 10 × 10 µm^2^ and a sandwiching structure of 30 nm Pt/30 nm SiO_2_ NRs/20 nm Pt. Thin Ag layers of 2 nm thickness were inserted between each electrode and SiO_2_ layer. It is well‐known that the Ag is suitable for implementing a volatile threshold switching device because of its low activation energy for diffusion and high mobility in SiO_2_ compared with commonly used Cu.^[^ ^36^, ^37^, ^38^, ^39^ ^]^ The 30 nm‐thick SiO_2_ NRs were deposited using a glancing angle deposition method (see Experimental section and Figure S1, Supporting Information). The plane and cross‐sectional scanning electron microscopy (SEM) images in Figure 2A show porous SiO_2_ NRs. For comparison, a device with a SiO_2_ thin film was deposited using an on‐axis mode. The well‐distinguishable sandwiching structures of the SiO_2_ thin film and NR devices were observed using SEM and transmission electron microscopy (TEM) images in Figure 2B and Figure S2, Supporting Information. Compared with the dark‐field scanning TEM (STEM) images of the dense SiO_2_ thin film shown in Figure S2C, bright spots appear in the SiO_2_ NRs, as shown in Figure S2F, Supporting Information, which can be attributed to the Ag nanoparticles or voids between the SiO_2_ NRs. The details are discussed in a later section.

Figure S3A–C, Supporting Information, shows typical threshold switching characteristics of a dense SiO_2_ film device including a large electroforming voltage of −13 V and long latency of 15 ms, which is attributed to the fact that the Ag‐based CFs are formed by the electromigration through the dense SiO_2_ matrix.^[^ ^32^, ^33^ ^]^ Therefore, we first attempted to understand the electrochemical metallization (ECM)‐based switching behaviors of our memristors depending on the porosity of the insulating SiO_2_ NRs. Regarding the DC electrical behavior of the 2 nm Ag/30 nm‐thick‐porous SiO_2_ NRs/2 nm Ag device in Figure 2C, the bidirectional threshold switching behavior was observed under voltage sweeps (from 0 to −2 or 2 V) with a compliance current (*I*_cc_) of 1, 10, and 100 µA. As the voltage increased to approximately 0.75 V (or −0.75 V), the device was turned on, and the current rapidly reached the *I*_cc_ level. After removal of the voltage sweep, the device automatically turned off spontaneously from its on‐state with a sudden current drop. The response of the device to the DC voltage sweeps represents the typical threshold switching behavior as demonstrated anywhere.^[^ ^33^, ^40^, ^41^ ^]^ Figure S3D, Supporting Information, shows the endurance characteristics of the device for 100 DC cycles without degradation. In addition, Figure S4, Supporting Information, shows uniform device‐to‐device switching characteristics including leakage current, threshold voltage, and volatile switching in ten different devices. To quantify the threshold voltages, statistical values were extracted from the 100 cycles *I*–*V* curves in Figure S3D, Supporting Information, and the average switching voltage is −0.72 and 0.78 V (Figure 2D). Figure 2E shows the statistical distributions of the electroforming voltage with an average of −0.83 V, which was extracted from 20 different cross points using the SiO_2_ NRs device. Compared with the threshold voltage, the subsequent switching voltages are similar to the first electroforming voltage, indicating that the device is more or less electroforming‐free. Furthermore, Figure 2F and Figure S5, Supporting Information, show 750 times shorter latency of 20 µs with a relaxation time of 15 µs for the 1, 2, and 3 nm Ag embedded SiO_2_ NR device compared with that of the SiO_2_ thin film device, indicating that the memristor latency is comparable to the biological channel opening of 40 µs. The repeatable pulse responses of the identical device and uniform pulse switching characteristics of five different devices were observed in Figures S6 and S7, Supporting Information, respectively. These unique electrical properties were considered to be related to preconditioned conductive paths and electromigration of Ag on the surface of SiO_2_ NRs.

The electrical characteristics of the threshold switching device are closely related to the formation and rupture of the CFs in the insulating layer of the metal‐insulator‐metal (MIM) structure.^[^ ^42^ ^]^ Accordingly, we investigated the influence of the structure on electrical switching characteristics. Figure 2G shows the electrical switching curves with the first electrical operation as a function of the Ag content from 1 to 10 nm between the electrode and the insulating layer. As the amount of Ag increased, the leakage current continuously increased and reached the compliance level for the 10 nm Ag device. This behavior is attributed to the pre‐dispersion of Ag on the surface in the SiO_2_ NRs during the fabrication process instead of being electrically driven into the oxide during the first operation. To investigate the effect of the Ag reservoir between the Pt electrode and SiO_2_ NRs, the switching characteristics of the device in which Ag was deposited only at the lower or upper interface of the SiO_2_ layer were examined (Figure S8, Supporting Information). Both interface Ag‐deposited devices show typical bidirectional switching curves, whereas the unidirectional switching with a higher threshold voltage was observed for the lower or upper interface Ag‐deposited device owing to the presence of a one‐side reservoir of the Ag atoms. These results imply that the threshold switching characteristic is attributed to the position of Ag reservoir. Furthermore, Figures S9 and S10, Supporting Information, show that the electroforming is independent of the SiO_2_ NR thickness up to 100 nm and is neglected with increasing SiO_2_ porosity. These are discussed in terms of the preconfigured Ag and the surface ionic migration in Note S1, Supporting Information.

**Figure 3:**
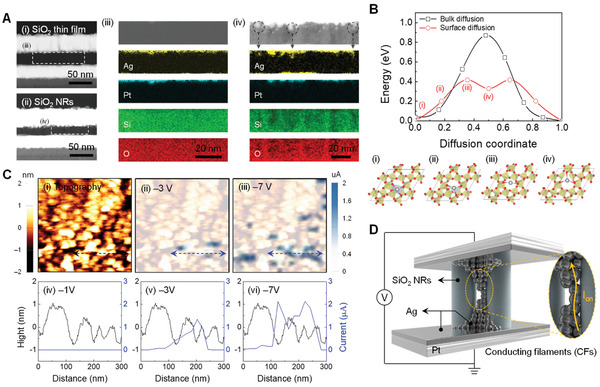
Threshold switching through surface electromigration of Ag. A) Cross‐sectional dark‐field STEM images of i) SiO_2_ thin film and ii) NRs memristors. The dotted area indicates a selected EDS mapping area. EDS mapping of Ag, Pt, Si, and O in the switching matrix of iii) SiO_2_ thin film and iv) NRs. B) Computed bulk and surface diffusion energy landscape for single Ag atom diffusion inside *α*‐SiO_2_ structures (upper figure). The diffusion path is from one tetrahedral site to another site inside the bulk or on [0001] oxygen surface. The computed surface diffusion path for Ag on [0001] oxygen surface of SiO_2_ (lower images). Red, yellow, and grey atoms indicate oxygen, silicon, and silver atoms, respectively. The migration transition state occurs when Ag atom is coordinated by only two oxygen atoms. C) i) Digitized topographic AFM image of a SiO_2_ NRs/Ag/Pt device. Overlapped topographic and current images at applied voltage of ii) −3 and iii) −7 V (upper images). Line profile of the topographic and current images at applied voltage of iv) −1, v) −3 V, and vi) −7 V (lower figures). D) Schematic illustration for threshold switching through the surface electromigration of Ag.

To verify the pre‐dispersed Ag on the surface of the SiO_2_ NRs, energy‐dispersive X‐ray spectroscopy (EDS) elemental analysis was performed in a selected area in cross‐sectional dark‐field STEM images (**Figure** **3**A(i,ii)). Figure 3A(iii,iv) show EDS images of the SiO_2_ thin film and NRs, respectively, where the upper panels are the selected dark‐field STEM images, and elemental Ag, Pt, Si, and O are indicated as yellow, cyan, green, and red dots, respectively. Compared with the STEM image of the SiO_2_ thin film, bright spots appear in the SiO_2_ NRs, as indicated by the dotted circles. Based on the EDS maps, the elemental Ag dots were consistent with the bright spots, whereas the elemental Pt did not diffuse into the SiO_2_ matrix. The lattice fringes in the interface and inside the SiO_2_ NRs are shown in Figure S11, Supporting Information. The diffraction patterns obtained by fast Fourier transform indicated cubic phase Ag. Therefore, these results imply that Ag was pre‐dispersed in the switching matrix during fabrication.

First‐principles calculations show that Ag diffusion is distinctively fast through the SiO_2_ surface compared to that through the bulk. Figure 3B shows the representative migration barriers computed for a single Ag atom in *α*‐SiO_2_ through bulk and surface diffusion. The migration barrier for bulk diffusion reached 870 meV, whereas that for surface diffusion was computed to be only 420 meV. The bulk Ag diffusion was modeled by diffusion from one tetrahedral site to a neighboring tetrahedral site (Figure S12, Supporting Information). The computed surface Ag diffusion path shows that the surface migration barrier is largely determined by the coordination number for the diffusing Ag atom, as shown in Figure 3B(iii). The initial Ag atom adsorbed on the SiO_2_ surface is located inside tetrahedral interstitial sites coordinated by four oxygen atoms. When diffusing out of the tetrahedral sites, the limited number of oxygen atoms available at the surface results in a reduced coordination number of three. The transition state involves the Ag atom coordinated only by two oxygen atoms forming 180° with each other, suggesting that the elevated energy is caused by insufficient coordination with oxygen atoms. This contrasts the bulk Ag diffusion with well‐coordinated interstitial diffusion and explains the facile Ag cation diffusion through SiO_2_ NRs (see Note S2, Supporting Information).

Our memristor performance was considered to be related to electromigration on the surface of the SiO_2_ NRs. To focus on the active switching area, which is supposed to be the location of the SiO_2_ NR surface and electrically conducting area, we performed conductive atomic force microscopy using a 30 nm‐thick SiO_2_ NRs/2 nm Ag/20 nm Pt device. The bottom Pt electrode was grounded, and the tip was negatively biased. In this case, Ag electromigration was initiated from the bottom Ag reservoir. The digitated topographic image in Figure 3C(i) shows that the locally recessed regions correspond to the voids induced by porous SiO_2_ NRs. By applying biases of (ii) −3 and (iii) −7 V, the locally conducting spots increase with sub‐µA conductance, as shown in the corresponding local current images overlapped with the topographic image. As shown in the line profiles (iv, v, and vi) of topographic and current images along the arrow in the upper images, the memristor is triggered above −3 V, exceeding a threshold value, and the high current paths are consistent with the locally recessed region in the SiO_2_ NRs. Therefore, it is highly probable that the Ag migrates along the surface of the SiO_2_ NRs.

Based on the results, we illustrate the ECM‐based threshold switching mechanism in the SiO_2_ NR memristor. Figure 3D shows vertical MIM structures with Ag clusters in the SiO_2_ NRs between the Pt electrodes. Based on the results of electroforming‐free and EDS elemental analysis, it can be assumed that such Ag clusters are present in the as‐fabricated state in the SiO_2_ NRs and are not generated by electrical stress. Under the application of an external bias above the threshold voltage, the nanoscale CF is formed by the surface electromigration of Ag atoms that electrically connect the two electrodes, leading to an electrical switching from HRS to LRS. After removal of the voltage sweep, the metallic CF can spontaneously break because of surface diffusion of Ag driven by the minimization of the surface energy.^[^ ^37^ ^]^

**Figure 4:**
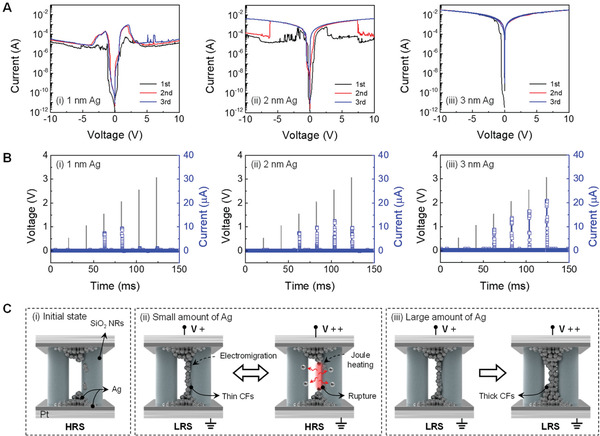
Selective response to specific amplitude range of stimuli. A) *I*–*V* curves of the threshold switching under DC voltage sweeps without compliance current as a function of Ag amount of i) 1 nm, ii) 2 nm, and iii) 3 nm. B) Pulse response of i) 1 nm, ii) 2 nm, and iii) 3 nm Ag embedded memristor with a train of 500 µs width with different amplitudes of 0.5–3 V. C) Illustration of a conduction mechanism. i) initial state. ii) Threshold switching with adaptation for the small amount of Ag. iii) Threshold switching with maladaptation for the large amount of Ag.

Selective response and electrical tunability are key biological functions for adaptation to an innocuous stimulus and maladaptation to a noxious stimulus. To design the diffusive memristor as its biological counterpart, we controlled the shape of the CF through the injected amount of Ag in the device. **Figure** **4**A shows DC voltage sweeps without a compliance current embedded with 1, 2, and 3 nm Ag. For the 1 nm Ag device (Figure 4A(i)), the set (from HRS to LRS) and reset (from LRS to HRS) transitions repetitively performed in identical polarity, referring to the unipolar switching characteristic induced by Joule heating in the CF at a high applied voltage. The on‐state continuously increased with the amount of Ag, and unipolar reset switching was not performed for the 3 nm Ag device (Figure 4A(iii)), implying the formation of a thick CF. Consequently, we can infer that the CF is finely controlled from thin to thick diameter through the amount of deposited Ag.

The tunable electrical characteristics of preconfigured Ag can be a great benefit in realizing a selective response to a specific range of stimulus intensity. Figure 4B and Figure S13, Supporting Information, show the pulse responses of the memristors by applying 500 µs pulse stimuli with different amplitudes of 0.5–3 V. It is worth noting that, during these single pulse measurements, a very long interval time (20 ms) was used in between, such that the device had enough time to relax back to its resting state, as shown in Figure 2F. With a single electrical pulse of 500 µs, the 1 nm Ag device was not turned on until the pulse amplitude reached 1.5 V, and a further increase of the amplitude to 2 V resulted in a large output in Figure 4B(i). Above the voltage of 2.5 V, the current does not increase. Compared with the 1 nm Ag device, a higher voltage is required for the 2 nm Ag device to initiate the decrease in the current (Figure 4B‐ii) and the output current for the 3 nm Ag device steadily increases (Figure 4B‐iii), indicating a selective response to their triggered amplitude range. The conducting mechanism for different amounts of Ag is summarized in Figure 4C. In the initial state shown in Figure 4C(i), pre‐dispersed Ag clusters are formed in the insulating SiO_2_ NRs to minimize the interface energy. For the small amount of Ag device in Figure 4C(ii), a thin Ag CF is formed when a pulse exceeding the threshold voltage is applied to the device. After the high amplitude voltage pulse is applied to the device, the thin CF can be ruptured by Joule heating from the thinnest region, enabling electrical switching from LRS to HRS, which is demonstrated by the thermochemical mechanism^[^ ^43^ ^]^ and corresponding to the results in Figure 4A(i),B(i). In contrast, the output current steadily increases in the case of a large amount of Ag device, as shown in Figures 4A(iii),B(iii) because of the abundant Ag reservoir depicted in Figure 4C(iii). These behaviors can be used to mimic a selective response to a specific amplitude range of external stimuli.

**Figure 5:**
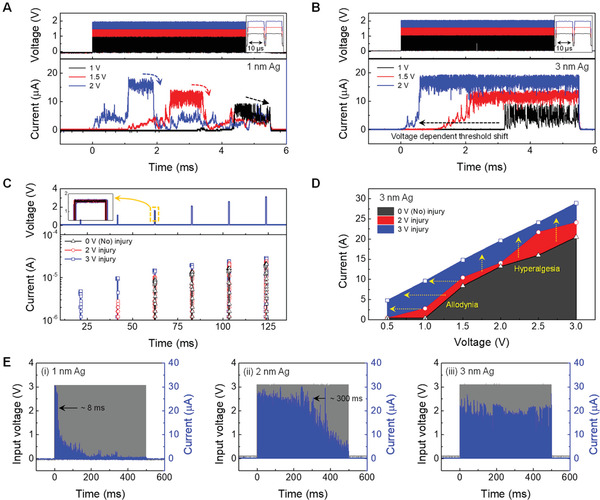
Electrical tunability for adaptive and maladaptive operation. Pulse response of A) 1 and B) 3 nm Ag memristors to multiple number of 10 µs pulses with different amplitude of 1–2 V. C) Train of input voltage pulses composed of variable pulse amplitudes of 0.5–3 V with a 500 µs pulse width applied on a nociceptor subjected to different injury pulses of 0, 2, and 3 V with a 500 µs width (upper panel) and the corresponding output currents (lower panel). D) Maximum output current at a different input voltage amplitude, demonstrating the shift of the ON‐switching voltage towards a lower threshold (Allodynia) and the On‐current towards higher current (Hyperalgesia). E) Pulse response of memristors to multiple number of 100 µs pulse width with amplitude of 3 V. The adaptation rate of i) 1, ii) 2, and (iii) 3 nm Ag memristors is classified as rapidly, slowly, and no‐adapting, respectively.

A train of electrical pulses was applied to the device to discuss the characteristics of our artificial receptors for prolonged stimulation. **Figure** **5**A,B show trains of 10 µs pulses of different amplitudes (1, 1.5, and 2 V, upper panel) and the corresponding output current pulses (lower panel) of 1 and 3 nm Ag embedded devices. In both memristors, the current jump is observed after a certain period, which becomes shorter with a higher amplitude of the pulses, indicating that the threshold is highly dependent on the applied voltage. This suggests that numerous pulses are needed to turn the device on if the amplitude is lower. It is noteworthy that the 1 nm Ag device in Figure 5A was turned off with a certain subsequent of pulses after the current jump, which can be a demonstration of an adaptation behavior attributed to Joule heating. In contrast, the output current for the 3 nm Ag device in Figure 5B is maintained without adaptation to a train of electrical pulses. This maladaptation is a representative response characteristic of a nociceptor that protect an injured area by continuously generating warning signals to noxious stimuli and enhancing pain sensitivity. This enhanced sensitivity can be characterized by hyperalgesia and allodynia, referring to an increased response to a normally painful stimulus and pain resulting from a normally innocuous stimulus, respectively. To demonstrate the nociceptive features of our memristor, we first applied pulses with high amplitudes (2 and 3 V, 500 µs width) to the 3 nm Ag devices, to introduce a change that mimics the injury or damage to the nociceptor system. The current response under different input voltages was recorded for devices that experienced different levels of damage, as shown in Figure 5C. Clearly, a higher output current occurs for the injured nociceptor, and the maximum output current at different input voltages is presented on a linear scale in Figure 5D. As the injury amplitude increases, the threshold voltage shifts toward the lower end, whereas the output current shifts higher. This result indicates that a smaller threshold voltage is required to turn on a more seriously injured device, reproducing the allodynia and hyperalgesia characteristics in the nociceptor.

To compare the adaptation rate of each memristor, a train of electrical pulses (100 µs pulse width, 1 ms period, 3 V amplitude) was applied to the device. Figure 5E shows the electrical responses of the 1, 2, and 3 nm Ag devices, respectively. It is noteworthy that a pulse width of 100 µs is sufficiently longer than the latency of the device, resulting in a current jump from the first stimulus. As the amount of Ag increased from 1 to 2 nm, the device required more stimuli to start the adaptation (Figure 5E(i,ii)), and the 3 nm Ag device shows a maintained current level without adaptation as a nociceptor (Figure 5E(iii)). To evaluate the repeatable adaptive behavior, DC sweeps and a train of pulses were subsequently measured using the 1 nm Ag device after the first adaptation behavior in Figure S14A, Supporting Information. Figure S14B,C, Supporting Information, show typical threshold switching curves and adaptive behavior, and Figure S15, Supporting Information, exhibits the repeatable operation in identical memristor, implying there is no permanent damage that could cause problems with the subsequent electrical switching operation. In addition, when the period between pulses increased from 200 µs to 2 ms, as shown in Figure S16, Supporting Information, the adaptation is delayed for the 1 nm Ag, whereas the 3 nm Ag device responds without adaptation regardless of the pulse period. This means that device adaptation corresponds to stimulus‐frequency dependence that describes slow adaptation with low frequency, an important function of the adaptive receptor in the bio‐system.^[^ ^44^ ^]^

**Figure 6:**
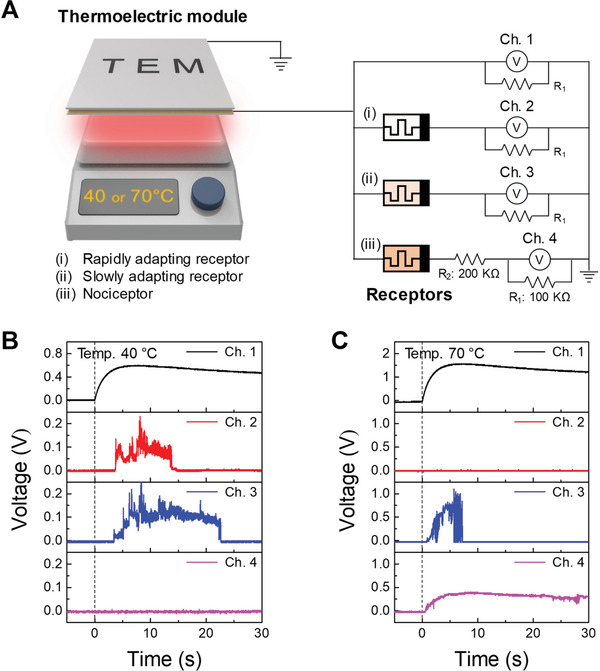
Artificial thermoreceptor using diffusive memristors. A) Schematic diagram of the circuit of an artificial sensory nervous system comprising a thermoelectric module and diffusive memristors. The generated voltage from the thermoelectric module and diffusive memristors monitored by oscilloscope channels at hot plate temperature of B) 40 °C and C) 70 °C. The monitored voltage in Ch. 1 is an external stimulus, and the voltages in Ch. 2, Ch. 3, and Ch. 4 indicate generated signals from the rapid, slow, and no‐adapting receptors.

Thermal perception is a fundamental physiological process pertaining to the vast majority of organisms.^[^ ^45^ ^]^ In vertebrates, the environmental temperature is perceived in the skin by primary afferents of somatosensory neurons. To demonstrate the potential application of our artificial receptors, we mimicked thermoreceptors in the human body. It is generally accepted that humans perceive temperatures of 15–45 °C as innocuous, and above this range as noxious.^[^ ^46^ ^]^ In this regard, the experimental temperatures were set to 40, 70, and 90 °C to mimic innocuous and noxious stimuli. The thermoelectric module can convert a temperature gradient into electrical power. As shown in **Figure** **6**A and Figure S17, Supporting Information, a thermoelectric module (LM‐5050‐3.7‐15.2, Livingcare) generates a voltage that is used as a stimulus signal when it is placed on the hot plate. The intrinsic characteristics of the thermoelectric module were monitored at Ch. 1 through direct connection with an oscilloscope, and diffusive memristors of 1, 2, and 3 nm Ag were connected in series with Ch. 2, 3, and 4, respectively. The generated voltage from the thermoelectric module is transmitted to each memristor. If the voltage exceeds the threshold value of the memristor, a series resistor senses the output voltage in the turned on channel. The resistance was carefully chosen to be 100 kΩ for Ch. 2, Ch. 3, and Ch. 4, such that the thermoelectric voltage dropped almost completely on the memristor alone before it was turned on and then dropped evenly on the resistor and memristor after it was turned on. In particular, an additional resistor (*R*_2_) of 200 kΩ was connected in series with an artificial nociceptor (3 nm Ag device) and Ch. 4 to shift the threshold voltage upward, which is consistent with the high threshold characteristic of a nociceptor. A series‐connected resistor‐dependent threshold voltage shift is demonstrated in Note S3 and Figure S18, Supporting Information. The thermoelectric module was placed on a hot plate from 0 s to generate a voltage. Figure 6B shows the voltage transients of each channel at 40 °C. It was discovered that innocuous stimuli at 40 °C triggered Ch. 2 and Ch. 3 with rapid and slow adaptive behaviors, respectively, and the stimulus could not turn on Ch. 4 because most of voltage is divided in series‐connected resistors (*R*_2_). For the temperature of 70 °C, as shown in Figure 6C, the high amplitude voltage allows Ch. 2 and Ch. 3 to adapt relatively quickly because of the above‐mentioned Joule heating, and substantial output signals were observed for Ch. 4, suggesting the onset of an on‐switching event. At the temperature of 90 °C in Figure S19, Supporting Information, the output signal was generated only from Ch. 4. The adaptation and maladaptation observed here are consistent with the results shown in Figures 4 and 5. In terms of the “selective response” property of a specific temperature range, the 1 and 2 nm Ag devices respond only to the innocuous temperature of 40 °C followed by adaptation, and the 3 nm Ag device is triggered only at a noxious temperature of 90 °C without adaptation. This system successfully achieved an artificial thermoreceptor with key features including adaptation, maladaptation, and selective response, demonstrating the capability of the memristor‐based artificial receptor as a biological counterpart.

## 3 Conclusion

We have constructed and demonstrated a new class of artificial receptors using a surface‐dominated diffusive memristor. The unique Ag dynamics on the surface SiO_2_ NRs allow them to mimic the adaptive and maladaptive operation of sensory receptors. Furthermore, the key parameters of the biological receptor, including “short‐latency”, “selective response”, and “tunability”, were realized using a diffusive memristor for the first time. We comprised a diffusive memristor‐based thermoreceptor using adaptive and maladaptive artificial receptors. The adaptive receptor exhibited a selective response to innocuous temperature with the adaptive operation. Under noxious temperature, the output current is not generated for the adaptive receptor, whereas the nociceptor is triggered with the maladaptive operation. This new artificial receptor potentially serves as the interface between external information and the internal nervous system, incorporable into all kinds of biomimetic sensory systems such as vision, tactile, auditory, gustatory, and olfactory, for a replacement of malfunctioning organs of human and component of the sensory system of humanoid robots.

## 4 Experimental Section

### Diffusive Memristor Fabrication

A Pt/Ag/SiO_2_/Ag/Pt/Ti multilayer was fabricated on a SiO_2_/Si substrate using standard photolithography and lift‐off processes. The 20 nm/40 nm‐thick bottom Pt/Ti electrode was e‐beam evaporated. The Ag/SiO_2_/Ag layers were deposited using e‐beam evaporator on the top of the bottom electrode. To deposit the SiO_2_ thin film and NRs, evaporation was performed at glancing angle of 0° or 70°, respectively, at a rotation speed of 25 rpm. When the initial nuclei grow, the self‐shadowing effect develops based on the incident angle of the vapor flux. Therefore, highly porous and ordered NRs were fabricated on the bottom electrode. Subsequently, the 30 nm‐thick top Pt electrode was evaporated at a glancing angle of 60° in the off‐axis mode to prevent diffusion of the top Pt due to the porous SiO_2_ layer.

### Device Characterization

The morphologies of the fabricated samples were observed using a field‐emission SEM (Inspect F) and TEM (Titan and Techni TEM). The TEM samples were prepared using a focused ion beam. Dark‐field STEM images were acquired, and energy‐dispersive X‐ray spectroscopy (EDS) was employed for further analysis. The electrical current‐voltage measurements were conducted using a 4155A semiconductor parameter analyzer (Keysight). The diffusive memristor was tested with bidirectional current‐voltage sweep measurements with a compliance current during the threshold switching process. For pulse measurement, a DSO‐X 3014A oscilloscope (Keysight) and a pulse AFG‐3102C generator unit (Tektronix) were used. An electrical bias was applied to the top electrode, while the bottom electrode was grounded.

### First‐Principles Calculations

All ab initio computations were performed using density functional theory calculations implemented in the Vienna ab initio simulation package.^[^ ^47^ ^]^ Perdew–Burke–Erzenhof pseudopotentials with the projector‐augmented wave method^[^ ^48^, ^49^ ^]^ were employed along with the generalized gradient approximation^[^ ^48^ ^]^ for the exchange‐correlation function. No Hubbard‐like U correction was employed in all the calculations. Crystalline *α*‐SiO_2_ was used, assuming that the diffusion behavior inside the amorphous SiO_2_ was highly similar to that inside the crystalline *α*‐SiO_2_, especially when comparing bulk and surface diffusion. To find the initial interstitial sites for Ag atoms inside and at the surface of SiO_2_, we utilized Voronoi analyzers as implemented in the PyCDT package.^[^ ^50^ ^]^ Nudged elastic band (NEB) methods^[^ ^51^, ^52^ ^]^ were employed to calculate the diffusion barrier, and the fixed lattice parameters of pristine *α*‐SiO_2_ were used throughout the NEB calculations. Linear interpolation of the ionic positions was used between the endpoints for the NEB calculations. The supercell consists of 24 SiO_2_ formula units for both the bulk and [0001] slab, with one Ag atom diffusing inside. K‐point grids of 3 × 3 × 3 were used for the endpoints and fully automatic K‐point grids with a length parameter of 25 for the NEB calculations.

## Conflict of Interest

The authors declare no conflict of interest.

## Supporting information

Supporting Information

## Data Availability

Research data are not shared.
