# Supplementary material for: Artificial Adaptive and Maladaptive Sensory Receptors Based on a Surface‐Dominated Diffusive Memristor
Source: Adv Sci (Weinh). 2021 Nov 27;9(4):2103484. doi: 10.1002/advs.202103484 (PMC8811822; doi:10.1002/advs.202103484)
Supplement: Supplementary file 1 — Supporting Information [file ADVS-9-2103484-s001.pdf]

© 2021 Wiley-VCH GmbH

ADVANCED SCIENCE
Open Access

Supporting Information

for *Adv. Sci.,* DOI: 10.1002/advs.202103484

Artificial adaptive and maladaptive sensory receptors based on a surface-dominated diffusive memristor

Young Geun Song, Jun Min Suh, Jae Yeol Park, Ji Eun Kim, Suk Yeop Chun, Jae Uk Kwon, Ho Lee, Ho Won Jang, Sangtae Kim, Chong-Yun Kang, and Jung Ho Yoon**

Supporting Information

Artificial adaptive and maladaptive sensory receptors based on a surface-dominated diffusive memristor

Young Geun Song, Jun Min Suh, Jae Yeol Park, Ji Eun Kim, Suk Yeop Chun, Jae Uk Kwon, Ho Lee, Ho Won Jang, Sangtae Kim, Chong-Yun Kang, and Jung Ho Yoon**

Dr. Y. G. Song, J. E. Kim, S. Y. Chun, J. U. Kwon, Prof. C. Y. Kang, Dr. J. H. Yoon
Electronic Materials Research Center
Korea Institute of Science and Technology (KIST)
Seoul 02791, Republic of Korea
E-mail: cykang@kist.re.kr, jhyoon@kist.re.kr

Dr. J. M. Suh, Prof. H. W. Jang
Department of Materials Science and Engineering
Seoul National University
Seoul 08826, Republic of Korea

Dr. J. Y. Park
Department of Materials Science & Engineering
Korea Advanced Institute of Science and Technology (KAIST)
Daejeon 34141, Republic of Korea

J. E. Kim, J. U. Kwon
Department of Materials Science and Engineering
Korea University
Seoul 02841, Republic of Korea

S. Y. Chun, Prof. C. Y. Kang
KU-KIST Graduate School of Converging Science and Technology
Korea University
Seoul 02841, Republic of Korea

H. Lee, Prof. S. Kim
Department of Nuclear Engineering
Hanyang University
Seoul 02841, Republic of Korea

Supplementary Note 1

Threshold switching depending on the thickness and porosity of SiO_2 NRs.

The threshold switching characteristics are originated from the electric field-induced active metal migration. In this regard, the thickness of the switching matrix between two electrodes determines electroforming and subsequent threshold switching characteristics in the diffusive memristor. To investigate the thickness dependent switching behavior, we fabricated memristors with different thicknesses from 10 to 100 nm SiO_2 NRs. The Ag was deposited from 1 to 10 nm with a constant 1:10 ratio. Figure S9 shows I-V curves and SEM images with different thicknesses of SiO_2 NRs. For the 10 nm-thick SiO_2 device (Figure S9A), the leakage current reaches the compliance level owing to the electrical connection between top and bottom electrodes in the extremely thin SiO_2 NRs. As the SiO_2 thickness increases, the memristors show typical bidirectional switching curves with a first electrical operation, as shown in Figures S9B and C. It is noteworthy that the electroforming and threshold voltages are independent of the SiO_2 NR thickness, which is attributed to the pre-dispersion of Ag and the rapid Ag migration along the surface of SiO_2 NRs under electrical stimulation. When the SiO_2 NRs reach 100 nm-thick, it shows unstable I-V switching curves with a relatively large electroforming voltage (Figure S9D). Considering the relatively thick SiO_2 NRs, it is highly probable that the Ag was insufficiently pre-dispersed and formed unstable CF in the switching matrix.

The porosity of SiO_2 NRs is determined through the deposited angle in an e-beam evaporator. To investigate the effect of SiO_2 porosity on the electrical switching characteristics, we fabricated SiO_2 NRs device deposited at 40°. Figure S10A exhibits typical threshold switching behavior under voltage sweeps with compliance current (I_{cc}) of 10 μA. Figure S10B shows statistic distributions of the electroforming voltage with an average of −2.17 V, which is extracted from 20 different cross points using the SiO_2 NR devices deposited at 40°. As the deposited angle of SiO_2 film increases, the electroforming is negligible, as shown in Figure

S10C, which is attributed to the surface electromigration of Ag in the voids between SiO_2 NRs

Supplementary Note 2

Computed bulk and surface diffusion path for Ag inside α-SiO_2.

The diffusion inside bulk SiO_2 involves rather well-coordinated interstitial diffusion. The transition state in bulk diffusion involves silver atoms coordinated by six oxygen atoms forming a severely distorted octahedral site (Figure S12). The configuration immediately before and after the transition state involves 5 and 8 oxygen atoms coordinating Ag. Yet, the computed Ag-O bond lengths are notably higher in average (2.65 Å) than those observed in naturally occurring Ag_2O (2.10 Å) computed with the same set of pseudopotentials. This also sharply contrasts the bond lengths observed in surface diffusion, with the average bond lengths of 2.04 Å at the transition state, 2.14 Å near the transition state, and 2.07 Å at the end sites. Noting that α-quartz or SiO_2 in general has a density of only 2.65 g/cm^3, the large free volume available inside SiO_2 does not necessarily provide fast diffusion. Rather, surface diffusion provides transition sites with matching Ag-O bond lengths, or equivalently suitably sized diffusion channels, and thus significantly faster diffusion compared to bulk with excess free space available. This trend is expected to persist in amorphous bulk SiO_2.

Supplementary Note 3

Selective response of 3 nm Ag embedded memristor for artificial nocicpetor.

A selective response is a key function in the biological receptor, referring to as a response of adaptation only in an innocuous stimulus and of maladaptation only in a noxious stimulus. The 1 nm Ag embedded memristor shows the selective response with adaptive operation in an innocuous stimulus, as shown in Figures 4B and 6B. However, the 3 nm Ag embedded memristor shows a comparable threshold switching characteristic with that of the 1 nm Ag

memristor, which hinders to implement an artificial maladaptive receptor (nociceptor) with the selective response. To achieve the diffusive memristor with the selective response, we connected additional resistor of 100 KΩ and 200 KΩ in series with the maladaptive receptor. Figure S18 shows the generated voltage from the thermoelectric module in the oscilloscope Ch. 1 and Ag 3 nm embedded diffusive memristor in Ch. 4. The red curve indicates the output voltage without the series resistor. The blue and magenta curves are the output voltages with a resistor of 100 KΩ or 200 KΩ in series, respectively. As the connected resistance increases at the innocuous temperature of 40 °C in Figure S18A, the electrical switching is delayed because of the voltage dependent threshold shift as shown in Figure 5B. When the 200 KΩ resistor is connected, the artificial maladaptive receptor is not turned on. Under the noxious temperature of 70 °C in Figure S18B, the output signal is immediately generated because of the high generated voltage from the thermoelectric module. This system indicates the selective response to a specific range of stimulus intensity depending on the artificial receptor.

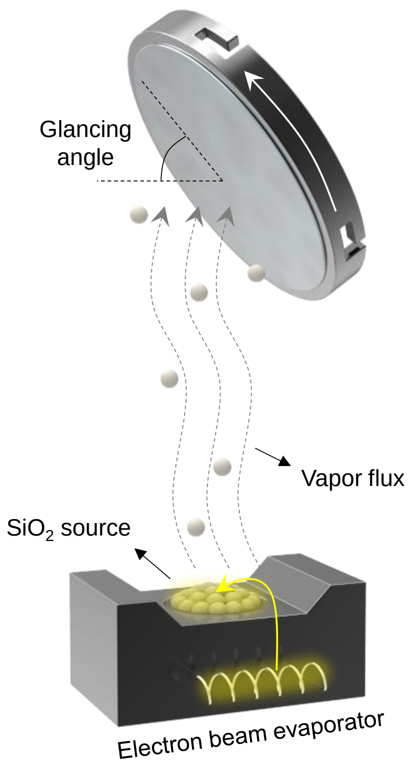

Figure S1. Schematic illustration of glancing angle deposition method using an electron-beam evaporator.

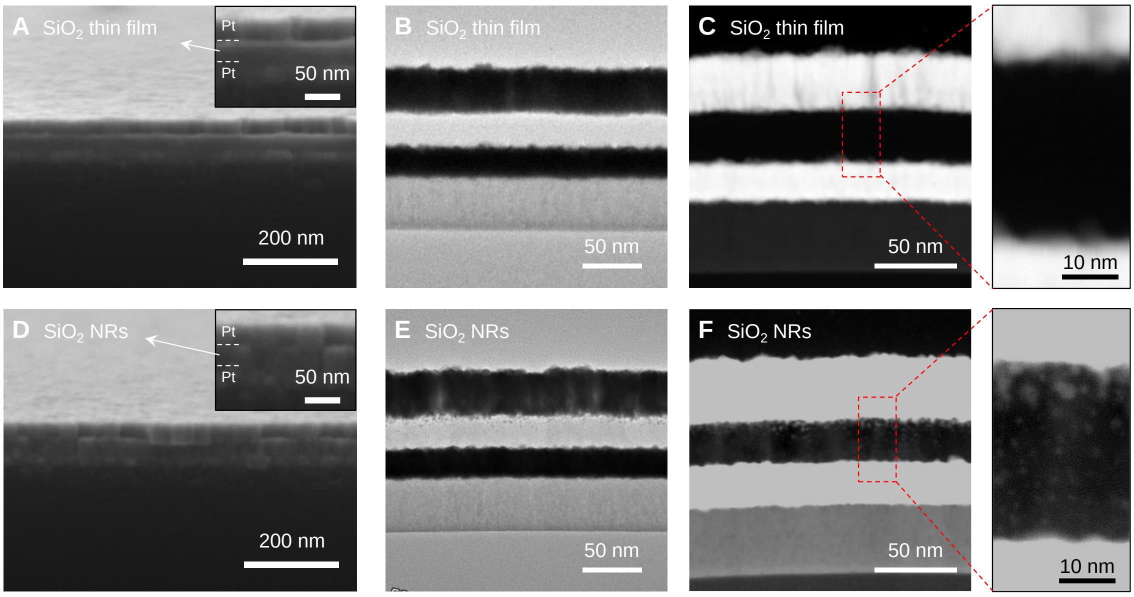

Figure S2. Cross-sectional view of the MIM structure. (A, D) SEM, (B, E) TEM, (C, F) dark-field STEM of Pt/SiO_2 thin film and NRs/Pt/Ti multilayer.

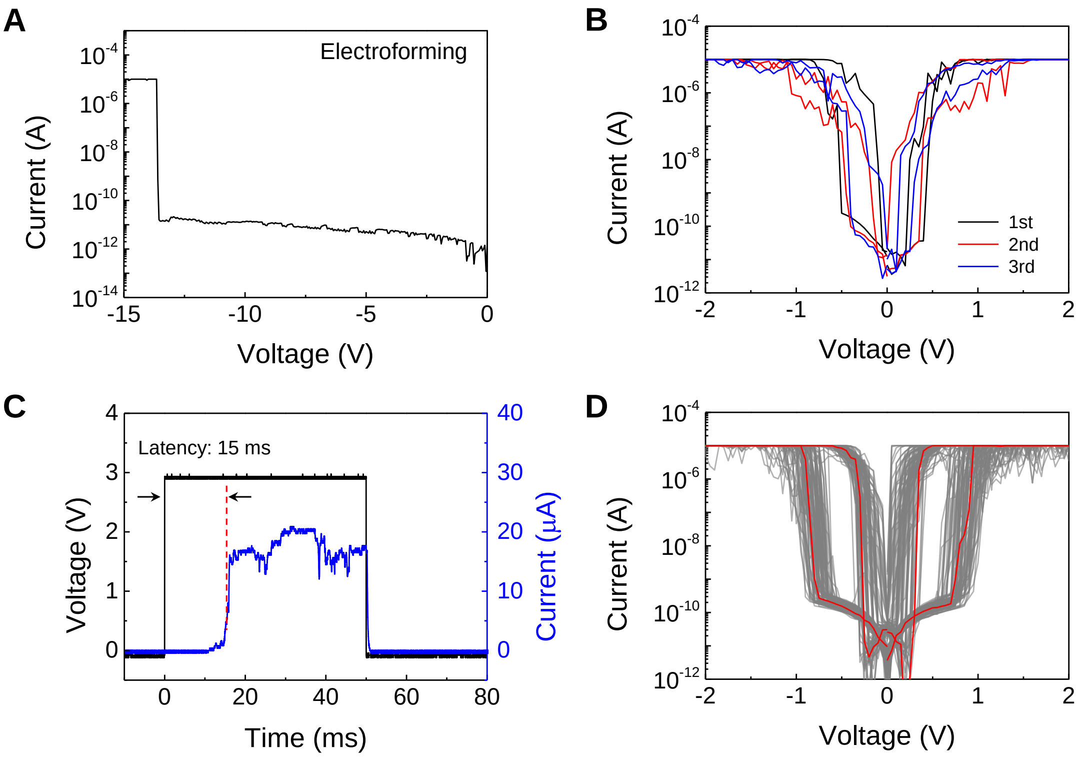

Figure S3. Threshold switching characteristics depending on Ag reservoir. A) Typical bidirectional I-V curves of both interface Ag deposited device. Unidirectional switching with higher threshold voltage of (B) lower and (C) upper interface Ag deposited devices. D) Uniform switching curves of SiO_2 NRs device for 100 cycles.

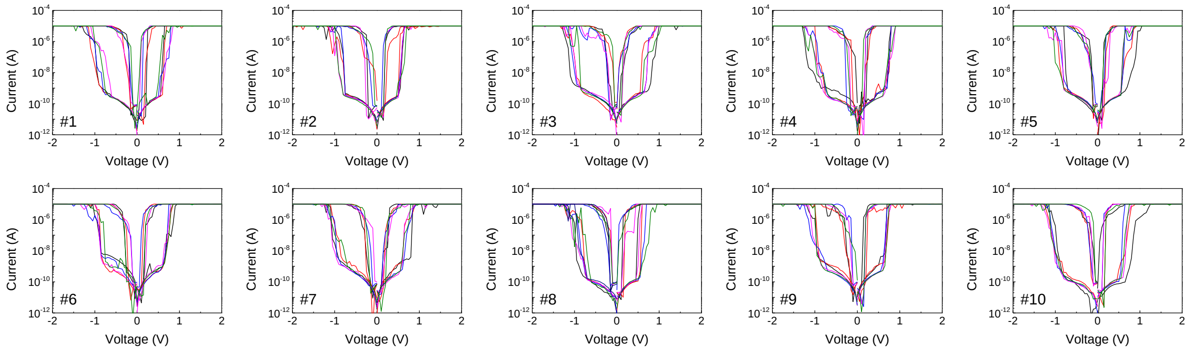

Figure S4. Threshold switching I-V curves of SiO_2 NR memristor with different 10 cross point.

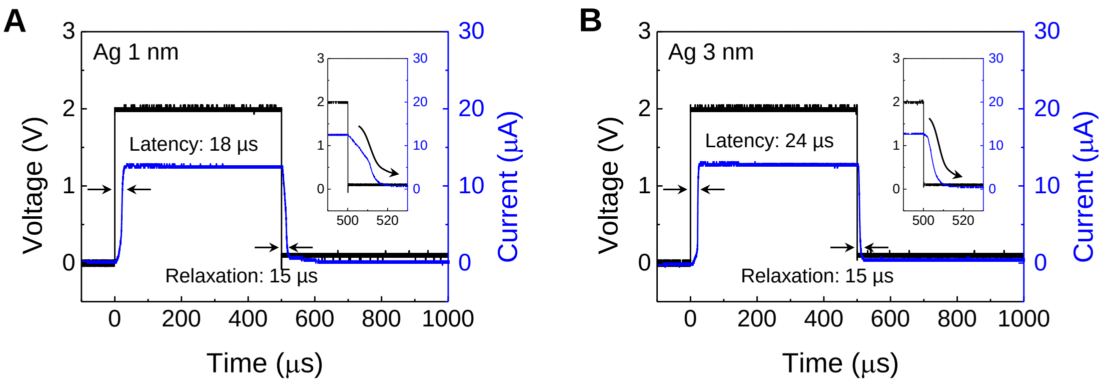

Figure S5. Latency and relaxation characteristics. Pulse response of (A) 1 nm and (B) 3 nm Ag embedded devices.

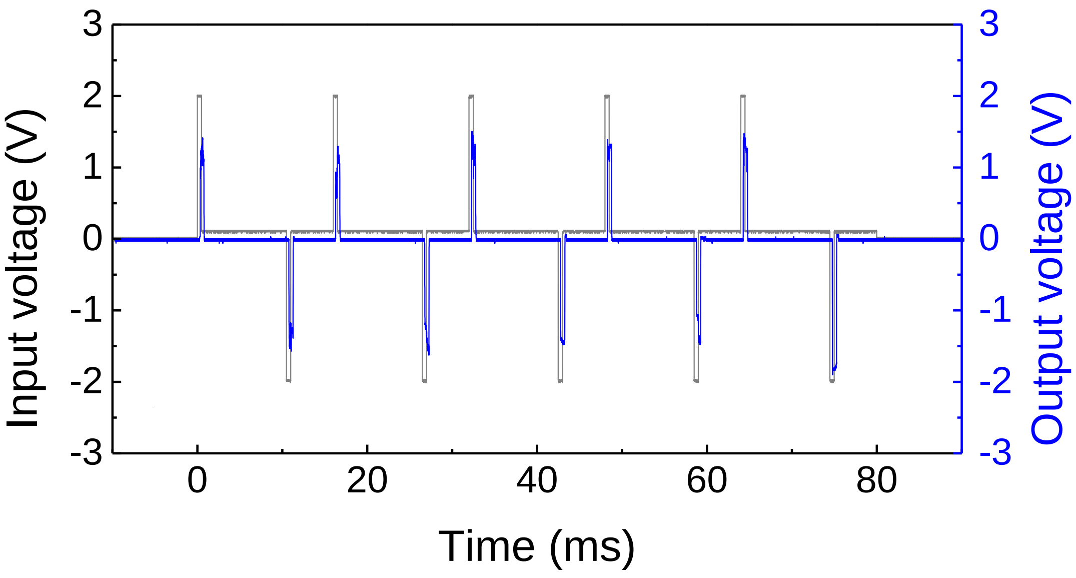

Figure S6. Repeatable pulse response of memristor towards a train of 500 μs with an amplitude voltage of 2 V and an offset voltage of 0.1 V.

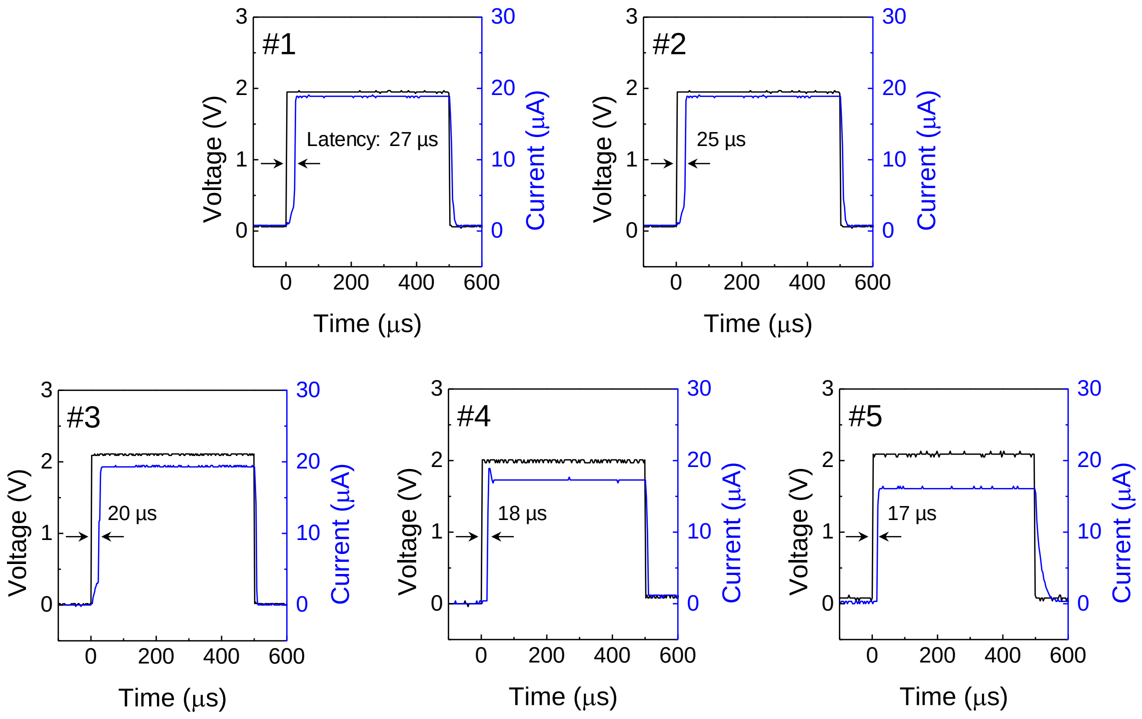

Figure S7. Pulse response of SiO_2 NR memristor with different 5 cross point.

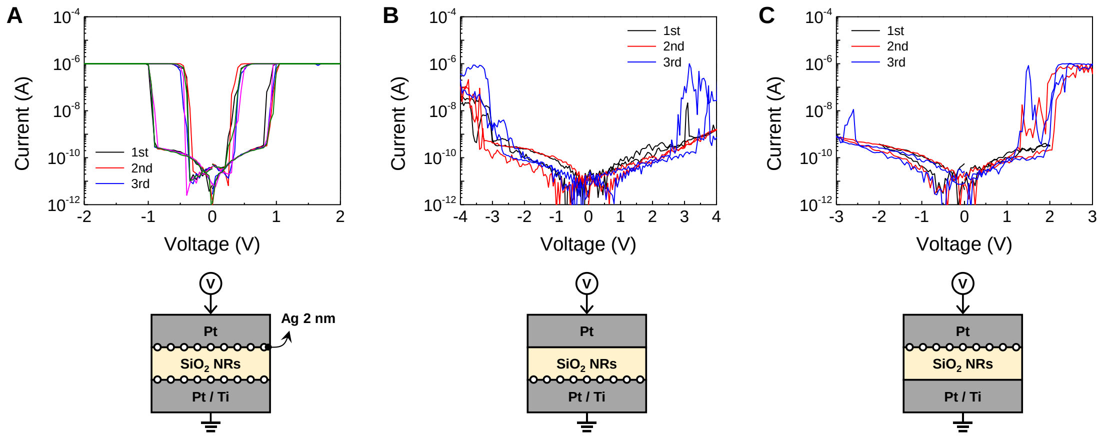

Figure S8. Threshold switching characteristics depending on Ag reservoir. A) Typical bidirectional I-V curves of both interface Ag deposited device. Unidirectional switching with higher threshold voltage of (B) Lower and (C) Upper interface Ag deposited devices.

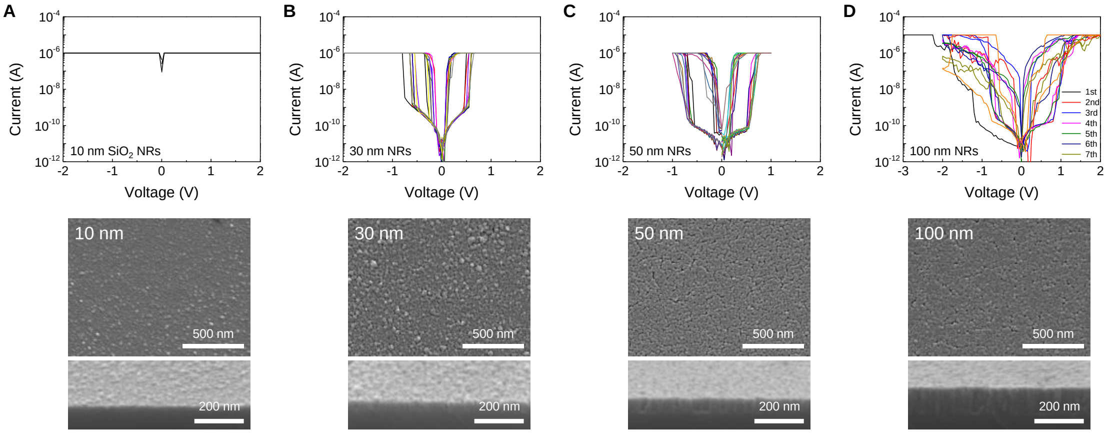

Figure S9. Threshold switching characteristics depending on thickness of SiO_2 NRs. Bi-directional I-V curves with different SiO_2 thickness of (A) 10, (B) 30, (C) 50, and (D) 100 nm.

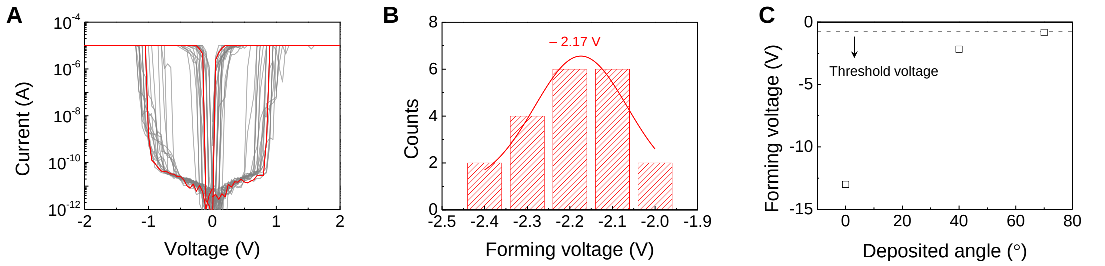

Figure S10. Electrical characteristics depending on deposited angle of SiO_2. A) Threshold switching I-V curves and (B) Histogram for the electroforming voltage distribution of SiO_2 NRs device with deposited angle of 40°. C) Electroforming voltage with different deposited angle of SiO_2 NRs using the glancing angle deposition method. The dotted line indicates the threshold voltage of SiO_2 NRs device with deposited angle of 70°.

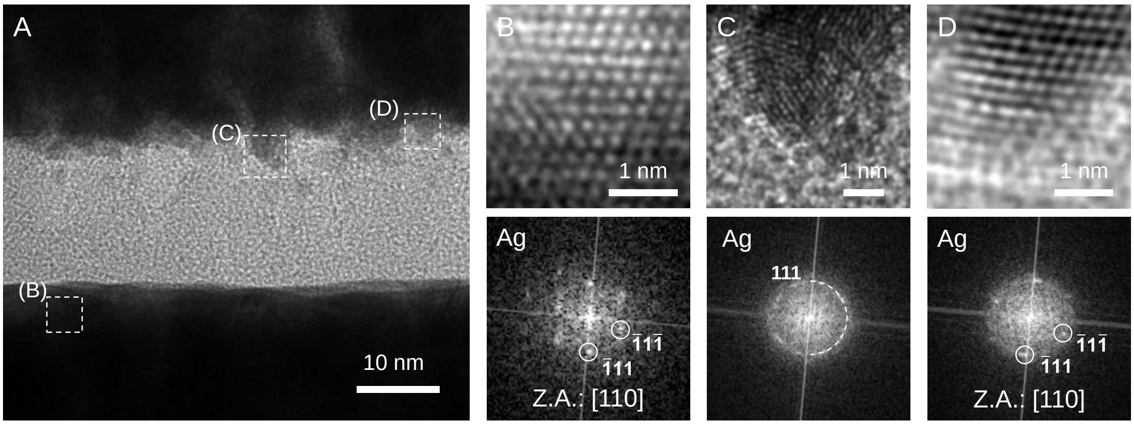

Figure S11. Distribution of the Ag in the switching matrix. A) Cross-sectional TEM image of the SiO_2 NRs based diffusive memristor. The 5 nm Ag was deposited at the upper and lower interfaces between SiO_2 and Pt. The lattice fringes corresponding to the left dotted boxes at (B) Lower interface, (C) Inside SiO_2 NRs, and (D) Upper interface. The diffraction patterns obtained from the upper lattice fringes by FFT.

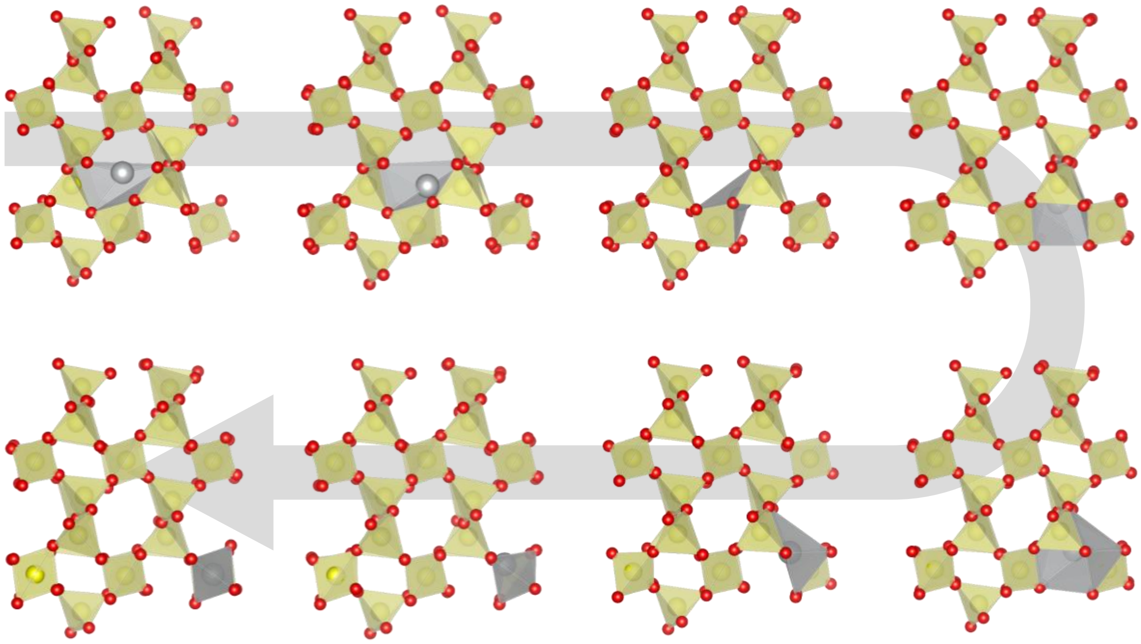

Figure S12. Computed bulk diffusion path for Ag inside α-SiO_2. Red, yellow and grey atoms indicate oxygen, silicon and silver atoms, respectively.

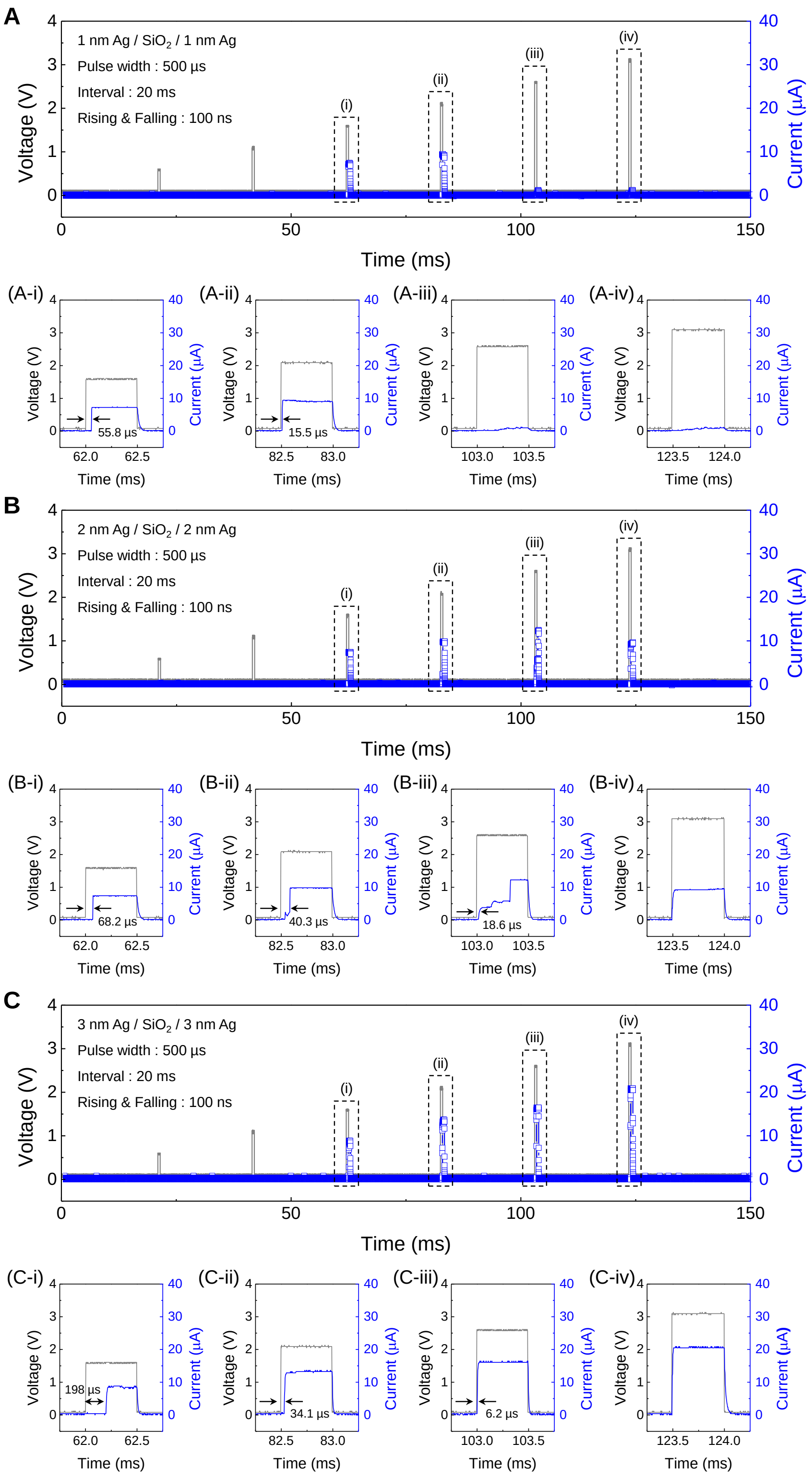

Figure S13. Zoomed-in pulse responses of the memristive receptors. Pulse responses of (A) 1 (B) 2, and (C) 3 nm Ag embedded memristors toward a train of 500 µs pulses with different amplitude of 0.5–3 V.

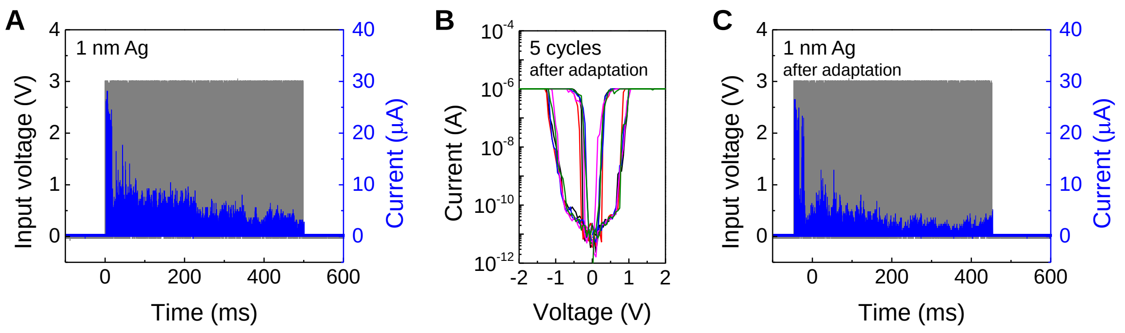

Figure S14. Repeatable adaptive operation. A) Adaptive response of 1 nm Ag embedded memristor to multiple number of 100 μs pulse width with amplitude of 3 V. Subsequent (B) DC sweeps and (C) adaptive response after the adaptive operation.

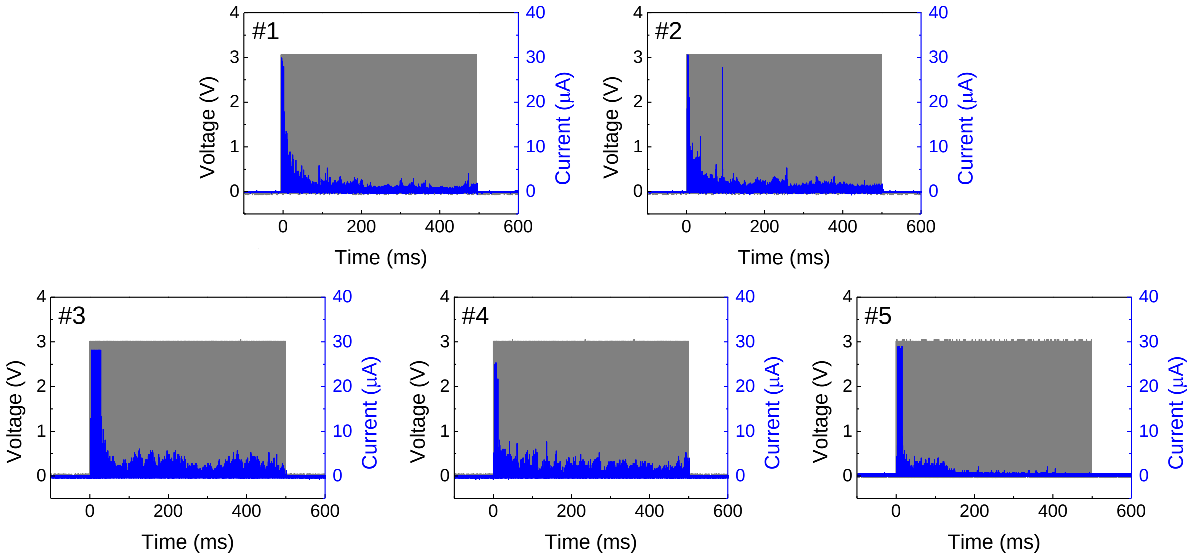

Figure S15. Repeatable adaptive operation of 1 nm Ag embedded memristor to multiple number of 100 μs pulse width with amplitude of 3 V.

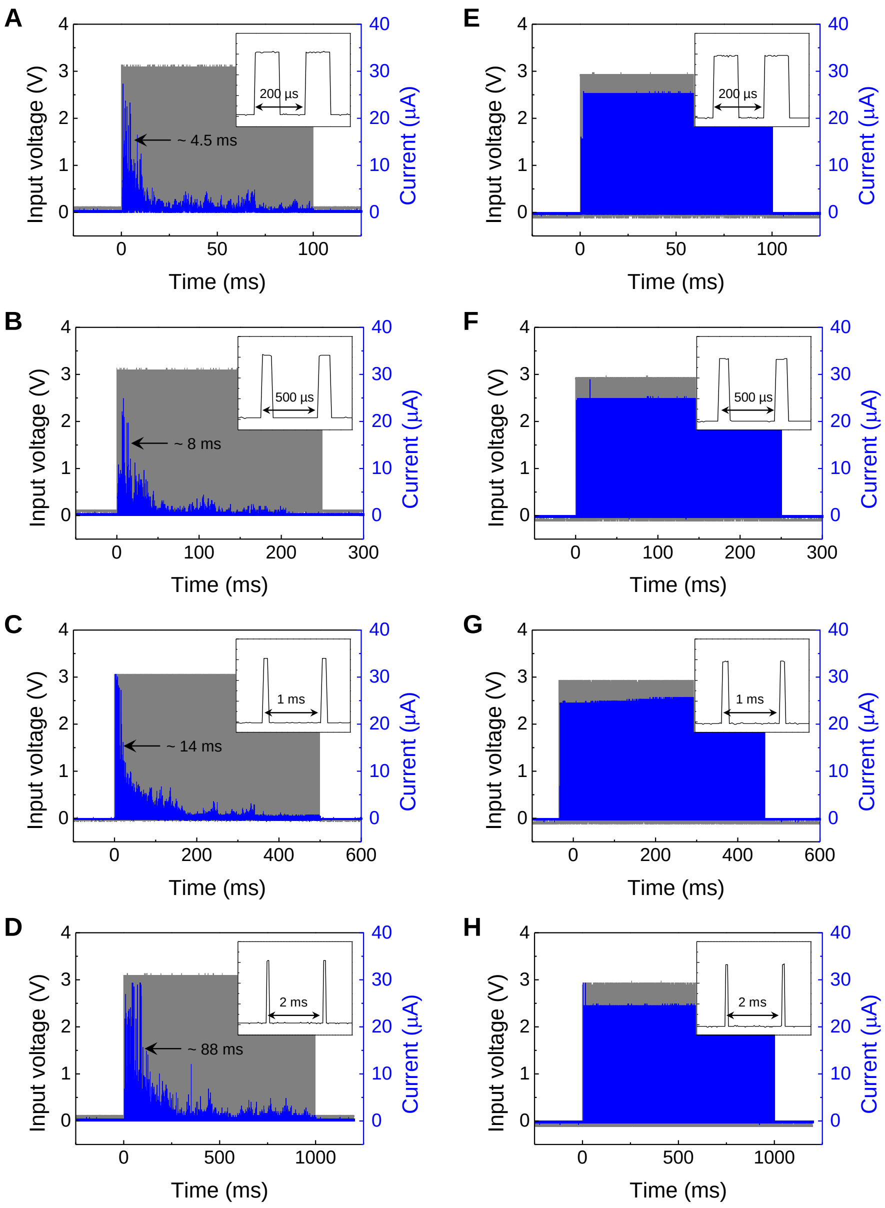

Figure S16. Adaptive and maladaptive operation toward different pulse period. Pulse responses of the (A–D) 1 nm and (E–H) 3 nm Ag embedded memristors to multiple number of 100 μs pulse width with amplitude of 3 V and various pulse period.

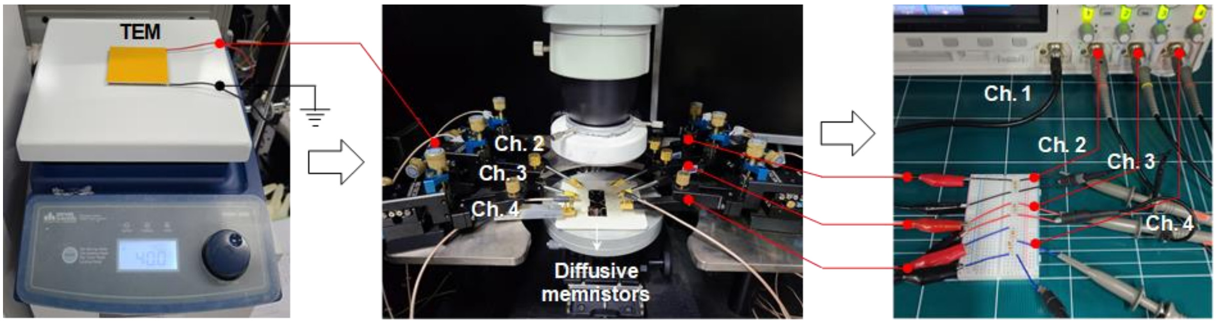

Figure S17. Experimental setup for artificial thermoreceptors using diffusive memristors.

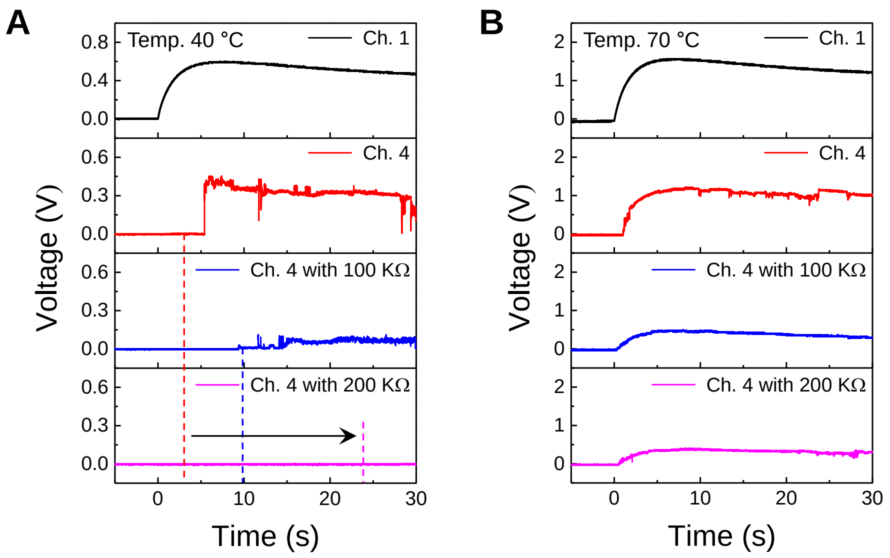

Figure S18. A series connected resistor dependent threshold voltage shift. Generated voltage from the thermoelectric module and Ag 3 nm embedded diffusive memristor with different series connected resistor at hot plate temperature of (A) 40°C and (B) 70°C.

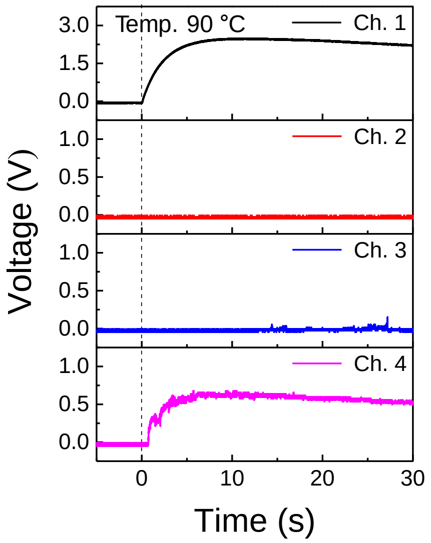

Figure S19. The generated voltage from the thermoelectric module and diffusive memristors monitored by oscilloscope channels at hot plate temperature of 90°C.
